# Impact of maternal prenatal stress by glucocorticoids on metabolic and cardiovascular outcomes in their offspring: A systematic scoping review

**DOI:** 10.1371/journal.pone.0245386

**Published:** 2021-01-22

**Authors:** Claudia Eberle, Teresa Fasig, Franziska Brüseke, Stefanie Stichling

**Affiliations:** Medicine with Specialization in Internal Medicine and General Medicine, Hochschule Fulda–University of Applied Sciences, Fulda, Germany; Texas A&M University College Station, UNITED STATES

## Abstract

**Background:**

“Stress” is an emerging problem in our society, health care system as well as patient care, worldwide. Especially by focusing on pre-gestational, gestational but also lactation phases “stress” is to be considered as an own trans-generational risk factor which is associated with adverse metabolic as well cardiovascular outcomes in mothers and their children. Hence, the maternal hypothalamic-pituitary-adrenotrophic (HPA) axis may be stimulated by various “stress” mechanisms as well as risk factors leading to an adverse *in utero* environment, e.g. by excess exposure of glucocorticoids, contributing to cardio-metabolic disorders in mothers and their offspring.

**Objective:**

To review the evidence of *in utero* programming by focusing on the impact of maternal “stress”, on adverse cardio-metabolic outcomes on their offspring later in life, by identifying underlying (patho-) physiological mechanisms (1) as well as adverse short and long-term cardio-metabolic outcomes (2).

**Methods:**

We conducted a systematic scoping review to identify publications systematically including reviews, interventional, observational, experimental studies as well as human and animal model studies. MEDLINE (PubMed) and EMBASE databases and reference lists were searched. Peer-reviewed articles from January 2000 until August 2020 were included.

**Results:**

Overall, n = 2.634 citations were identified, n = 45 eligible studies were included and synthesized according to their key findings. In brief, maternal hypothalamic-pituitary-adrenotrophic (HPA) axis might play a key role modifying *in utero* milieu leading to cardio-metabolic diseases in the offspring later in life. However, maternal risk factor “stress”, is clearly linked to adverse cardio-metabolic offspring outcomes, postnatally, such as obesity, hyperglycemia, insulin resistance, diabetes mellitus (DM), Metabolic Syndrome (MetS), cardiovascular disease (CD), hypertension, restricted fetal growth as well as reduced birth, adrenal, and pancreas weights.

**Conclusions:**

Women who experienced “stress” as risk factor, as well as their offspring, clearly have a higher risk of adverse short- as well as long-term cardio-metabolic outcomes. Future research work is needed to understand complex transgenerational mechanisms.

## Introduction

Diabetes mellitus, obesity, Metabolic Syndrom (MetS), but also cardiovascular diseases (CD) emerge worldwide [1]. Causes are assumed to be, e.g. changes in life style factors, such as high carbohydrate and/or high fat intakes as well as lack of exercises leading to, e.g. obesity, insulin resistance, T2D, hypertension and CD [1]. Meanwhile there is great evidence that maternal peri-gestational health, which is also affected by environmental as well as personal conditions, is linked to cardio-metabolic health of their offspring later in life [1].

The maternal risk factor “stress” is an emerging concern in maternity care and “stress” seems to be considered as an own risk factor in terms of transgenerational cardio-metabolic programming [1, 2]. Therefore, we analyze the evidence of *in utero* programming by focusing on the impact of maternal “stress”, peri-gestationally, on adverse cardio-metabolic outcomes on their offspring later in life, by identifying underlying (patho-) physiological mechanisms (1) as well as adverse short and long-term cardio-metabolic outcomes (2).

From a pathophysiological point of view, “stress” is defined as a condition of threatened homeostasis caused by intrinsic or extrinsic stressors that is redressed by a range of physiological and behavioral responses (“stress system”) to restore the optimal balance [3, 4]. The stress system mainly consists of the hypothalamic-pituitary-adrenal (HPA) axis and the autonomic nervous system (ANS). Glucocorticoids are the end products of the HPA axis. They play a key role in the perpetuation of resting and stress-related homeostasis affecting the adaptable reaction of the organism towards stressors. In terms of dysregulation of the stress response, allostasis has adverse effects on development, immune response, metabolism, and other physiological functions [3, 4]. In the following, we refer to this definition of stress.

Interestingly, pathophysiological changes of the *in utero* milieu may predict individualized developments in the offspring [5]. Two mechanistic hypotheses are widely accepted to explain the association between an adverse prenatal environment and postnatal health outcomes: fetal malnutrition (including both undernutrition and overnutrition) and fetal overexposure to glucocorticoids or stress [1, 6–8]. These hypotheses are intimately connected, meaning that the effects of nutritional insults on health may vary as a function of stress and conversely that the effects of acute or chronic stress may vary as a function of nutritional status [9]. Increased stress negatively affects maternal food intake resulting in a reduced nutrient delivery to the fetus [6, 7].

By provoking stress responses in the mother and fetus, nutritional manipulations cause alterations in fetal glucocorticoid exposure [7, 8]. Moreover, stress and its related biological processes have the ability to influence not only eating habits (e.g. caloric intake and selection of food types) but also metabolic fate of energy [9]. Since nutritional insults and stress are known to co-occur in many societies, both seem to play a key role in perinatal programming of body composition and cardio-metabolic function, and stress biology may represent the underlying mechanism [9].

Nevertheless, the majority of studies investigating the impact of adverse early environment have focused on the role of maternal nutrition during pregnancy [10], but psychosocial stress exposure during pregnancy attracts more and more attention [11]. For example, maternal stress can appear as depression, personal trauma, or self-perceived stress [12]. Stress is not only a factor which may again lead to mental disorders [13], but also to stress-related (patho-) physiological processes that have been implicated in a multitude of developmental health outcomes [1, 9]. Prenatal exposure to maternal stress may affect both a range of short-term outcomes, including size at birth as well as long-term outcomes affecting later life, such as altered behavior but also an increased risk of cardio-metabolic disease [11, 12].

This systematic scoping review provides a current overview of existing evidence regarding (patho-) physiological mechanisms underlying maternal stress exposure during pregnancy and its consequences on offspring health measures. In detail, we aimed at reviewing the existing evidence by identifying underlying mechanisms of maternal stress in *in utero* cardio-metabolic programming (1), and different adverse short and long-term cardio-metabolic outcomes in offspring (2), including reviews, interventional, observational, experimental studies as well as human and animal model studies.

## Materials and methods

### Information source and search strategy

We conducted a systematic scoping review to outline key (patho-) physiological mechanisms, examine different health outcomes, identify evidence gaps, and review different types of evidence [14]. A systematic scoping review follows the steps of a systematic review but with a broader scope [14]. We followed PRISMA for systematic reviews [15] and Joanna Briggs Institute for systematic scoping reviews guidelines [14].

EMBASE and MEDLINE via PubMed databases were systematically searched to identify studies published from January 2000 to August 2020. We followed PRISMA guidelines for systematic reviews (S1 Appendix). Search terms were: (cortisol) OR (glucocorticoids) OR (stress) AND (fetal development) OR (prenatal) OR (perinatal programming) AND (cardiovascular disease) OR (metabolic disease) as Medical Subject Headings and Embase Subject Headings terms and title/abstract terms (S2 Appendix). No protocol has been published. Studies were selected by two independent reviewers. First, we searched databases, eliminated duplicates, and selected publications by screening titles and abstracts. Second, we assessed studies with full-text for eligibility. Third, to identify further relevant evidence, reference lists of included studies were manually searched.

### Eligibility criteria

To increase our comprehensiveness, we included different study designs. Articles included were literature reviews and reports, interventional studies (randomized controlled trials, non-randomized controlled clinical trials), observational studies (cohort studies), and experimental studies. We included peer-reviewed articles with full-text published over the past 20 years (January 2000 until August 2020), and human and animal model studies in English and German. Eligible for review were publications examining the impact of maternal “stress” on *in utero* programming of adverse cardio-metabolic outcomes. As already mentioned, “stress” was defined from a pathophysiological point of view as a condition of threatened homeostasis caused by intrinsic or extrinsic stressors and redressed by a range of physiologic and behavioral responses that restore the optimal balance [3, 4]. We included environmental, physiological, and emotional stressors. Conference abstracts, letter, notes, and comments were excluded.

### Data extraction and synthesis

Study characteristics (authors, title, year of publication, design, objective), methods (e.g. intervention details, sample, patient characteristics), outcomes, main findings, and conclusions were extracted. Studies were grouped according to their key findings (mechanisms and outcomes) and our goals: (patho-) physiological mechanisms in perinatal cardio-metabolic programming (1), and the impact on short and long-term cardio-metabolic outcomes in offspring (2).

In accordance with the guidelines for (systematic) scoping reviews, we aimed at providing an overview of the existing evidence and reviewing different types of evidence regardless of quality [14]. Therefore, a formal quality assessment was not performed.

## Results

### Overview

**Fig 1:**
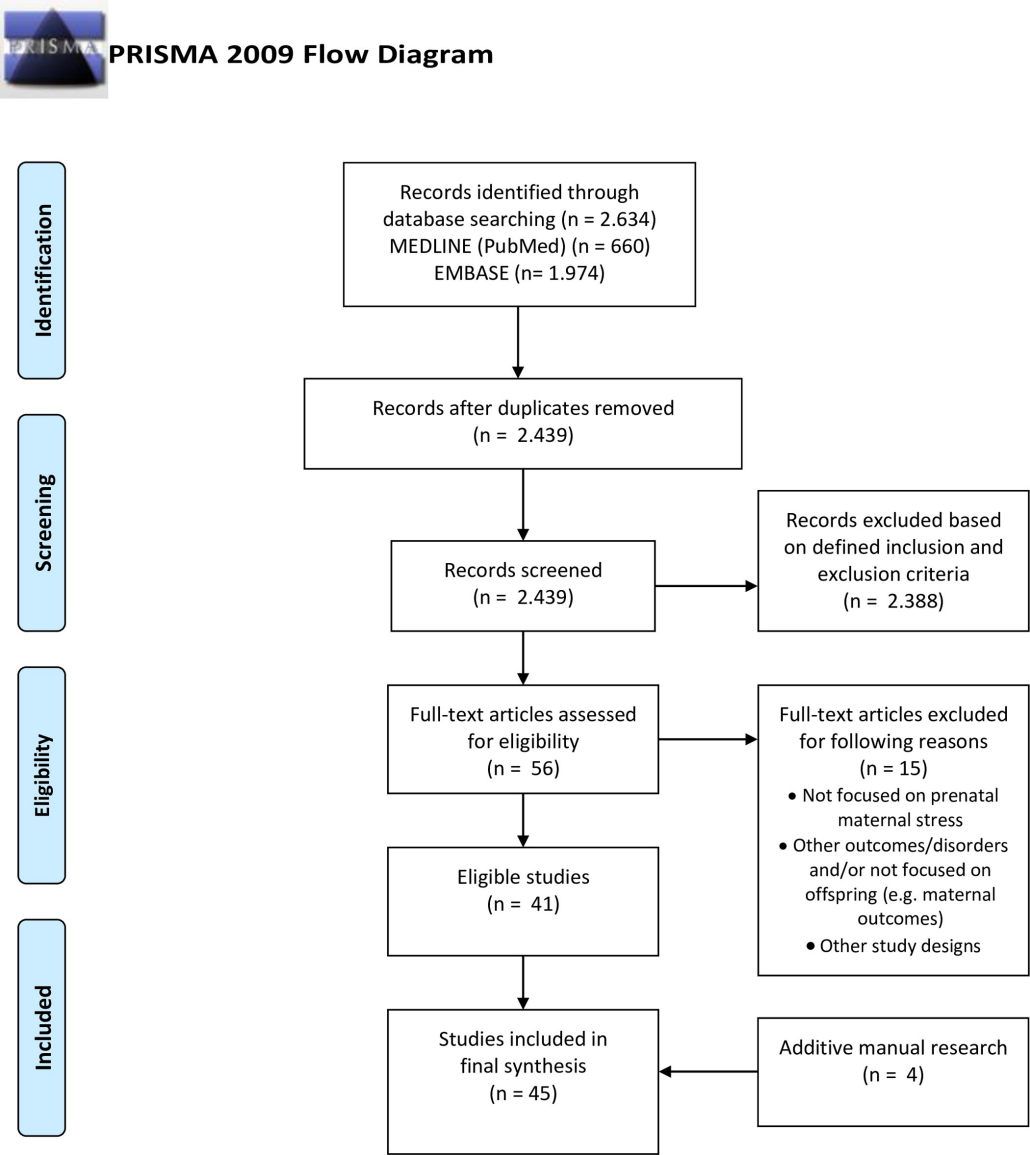
PRISMA flow diagram.

Search identified n = 2.634 citations. After removing duplicates, we screened n = 2.439 titles/abstracts. Excluding n = 2.388 ineligible articles based on inclusion and exclusion criteria, n = 51 publications remained. After removing n = 10 unsuitable full-text articles and identifying n = 4 studies [16–19] via manual research of reference lists, we finally included n = 45 publications in our synthesis. The search and selection process is displayed in PRISMA flow diagram (Fig 1). We identified n = 16 reviews, n = 12 cohort studies, n = 11 experimental animal model studies, n = 2 non-randomized controlled clinical trials, n = 1 randomized controlled trial, and n = 3 reports. An overview of the studies is provided in S3 Appendix.

### The key role of the hypothalamic-pituitary-adrenotrophic (HPA) axis in trans-generational programming of cardio-metabolic diseases

In a nutshell, the stimulation of maternal hypothalamic-pituitary-adrenotrophic (HPA) axis might play a key role modifying *in utero* milieu leading to cardio-metabolic diseases in the offspring later in life. Since there are no direct neural connections between the mother and the fetus, e.g. maternal psychological functioning has to be translated into physiological effects. At least three mechanisms are postulated how maternal psychological stress might have a direct impact on the fetus: alteration in maternal behaviors (e.g., substance abuse), reduction in blood flow leading to fetal deprivation of oxygen and nutrients, and transport of stress-related neurohormones from the mother to the fetus through the placenta [20].

During gestation the placenta functions as an exchange site between the mother and the developing fetus [11, 21]. This unique organ is supposed to modulate and filter signals from the maternal to the fetal milieu [8]. From a neuroendocrine and epigenetic point of view, there is evidence suggesting that the placenta is highly susceptible to maternal distress and thereby serves as a key mechanistic link between maternal distress and adverse offspring outcomes. Prenatal maternal distress may act through the placenta and in this way affect the growth and development of the fetus [11]. Maternal cortisol may be passed to the fetus through the placenta, generating a cascade of effects with potential impact on fetal development and offspring’s future health [2, 22–24].

**Fig 2:**
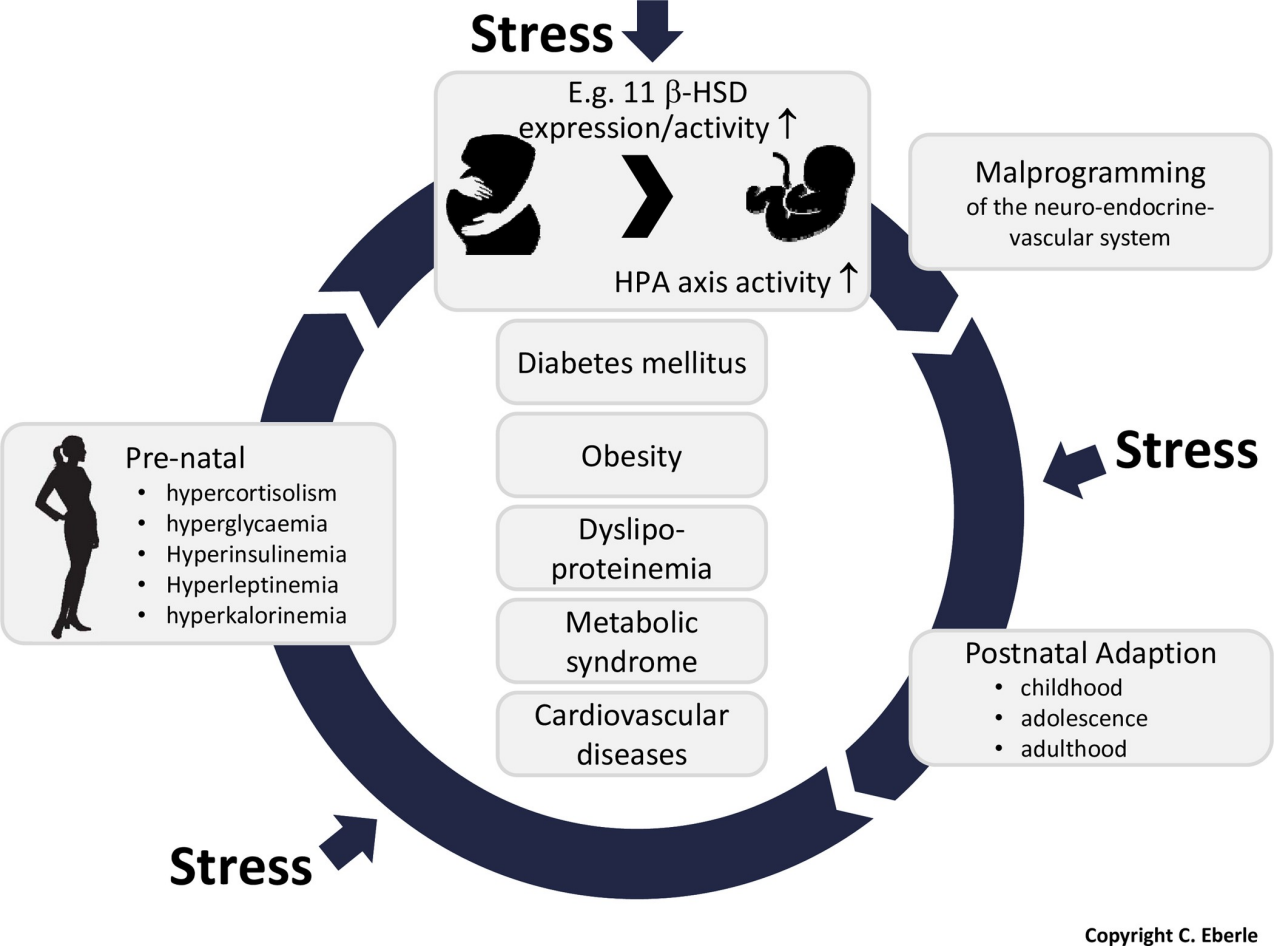


High levels of maternal glucocorticoids may cause altered gene expression profiles in placental tissues (Fig 2). One of these target genes is 11β-HSD2 encoding the enzyme 11β-hydroxysteroid dehydrogenase type 2. 11β–HSD2 is highly expressed in the placenta and functions as a protective feto-placental barrier to maternal glucocorticoids [8, 11]. Although glucocorticoids are able to cross the placenta, their levels are significantly lower in the fetus than in the mother because 11β–HSD2 can inactivate glucocorticoids. It catalyzes the rapid conversion of active cortisol to biologically inactive cortisone and thereby protects glucocorticoid sensitive tissues in the fetus from high levels of circulating maternal stress hormones [7, 11]. 10–20% of maternal glucocorticoids reach the fetus in its intact form indicating that this barrier is apparently incomplete. Compared to the fetus, maternal glucocorticoid levels are much higher. Only modest changes in the expression of placental 11β–HSD2 may profoundly influence fetal glucocorticoid exposure [11]. It is additionally postulated that reduced levels of placental 11β–HSD2 enable greater glucocorticoid signaling within the placenta itself, which can indirectly impact fetal development by changing placental function [7].

In mammals, glucocorticoids play a crucial role during fetal development. They are essential for growth regulation, tissue development and maturation of various organs. This action is critical to prepare the fetus for extra-uterine existence [6, 8]. Glucocorticoids exert their most potent effects during late gestation by stimulating surfactant production by the lung. For this reason, synthetic glucocorticoids are widely used to treat women at risk of preterm labor where immaturity of the lung highly impacts neonatal viability [6, 25, 26]. Antenatal glucocorticoid therapy can reduce birth weight which is associated with numerous long-term effects on the offspring’s health including an increased risk of cardio-metabolic diseases in adult life [6, 26].

Since 11β-HSD2 allows passage of a small amount of active glucocorticoids from the mother to the fetus, elevations in maternal cortisol can still double the amount of intact cortisol reaching the fetus [8, 22, 27]. Excess glucocorticoids reduce placental 11β-HSD2 expression and/or activity leading to an increased transport of active cortisol across the placenta. This is correlated with a lower birth weight, higher blood pressure and glucose intolerance in later life [6]. As an endproduct, one of the body’s major stress responsive system, glucocorticoids have been proposed to play a crucial role in stress-related perinatal programming of cardiovascular and metabolic disorders [2]. In this way prenatal stress/excess glucocorticoid exposure links fetal development to adverse adult health outcomes [27].

The HPA axis is a key part of the neuroendocrine stress response. In humans and many other species, HPA axis is activated in reaction to physical as well as psychological stress [22]. Hypersecretion of cortisol can permanently modify HPA function, causing an increased secretion of cortisol which in turn impacts development of the fetal HPA axis [28]. Evidence comes from studies in humans where high levels of prenatal psychosocial stress during pregnancy were associated with altered HPA axis functioning in adult life. Psychosocial stress was defined as multi-component construct that involves negative life events, appraisal of the stress, and symptoms such as anxiety. Impaired regulation of the offspring’s HPA axis regulation is linked to several adverse health outcomes including the metabolic syndrome [29]. Besides chronic stress conditions, maternal exposure to synthetic glucocorticoids and nutrient restriction can also significantly influence HPA function, leading to increased fetal exposure to glucocorticoids. Both is linked to cardiovascular diseases, insulin resistance and diabetes in later life [30, 31]. Birth weight of rats was reduced after prenatal exposure to the synthetic glucocorticoid dexamethasone or the 11β-HSD2 inhibitor carbenoxolone. During adulthood, the offspring display increased HPA axis activity, hypertension and hyperglycaemia [25]. Additionally, rodent experiments revealed that programming of HPA function can be passed to subsequent generations. Treatment of a pregnant rat with the synthetic glucocorticoid dexamethasone reduced offspring’s birthweight and glucose tolerance. These effects were transmitted to the grandchild generation without further exposure of the F1 generation [6, 32, 33].

Glucocorticoids exert their effects on the developing fetus by binding to glucocorticoid receptors (GRs) which subsequently act as transcription factors to alter gene expression levels [7]. GRs are expressed in most fetal tissues, including a high expression level in the placenta [27, 34] of the fetus to either high levels of stress or glucocorticoids can permanently affect GR expression [35]. In both animal and human studies, excessive production of endogenous glucocorticoids as well as their exogenous administration have been shown to cause effects on many systems such as diabetogenic and hypertensive effects [34].

Prenatal glucocorticoid administration to women having an increased risk of preterm delivery is associated with higher systolic and diastolic blood pressures in adolescents 14 years of age [36]. Antenatal glucocorticoid therapy is also linked to higher plasma insulin concentrations in subjects 30 years of age, which might result in insulin resistance later in life [37]. A prospective cohort study by Goedhart et al. analyzing n = 2.810 pregnant women indicated that high maternal cortisol levels measured in the morning were negatively associated to offspring birth weight and positively to being born small for gestational age [38]. In the Hertfordshire study, high cortisol levels in a cohort of elderly men and women were related to low recorded birth weights and a variety of cardiovascular risk factors, e.g. increased plasma glucose and triglyceride levels and increased systolic blood pressure [26, 39]

Furthermore, studies in several animal models have shown that excessive exposure to prenatal glucocorticoid excess reduces birth weight and subsequently causes hypertension and hyperglycemia in later life of the offspring [12, 34]. In rats, the correlation between low birth weight and adulthood diseases such as high blood pressure could be related to an adverse glucocorticoid environment *in utero* [31].

Administration of cortisol to sheep early in pregnancy elevates blood pressure in adult offspring [40, 41]. In rats, prenatal exposure to glucocorticoids during the last week of pregnancy was able to produce permanent effects by causing long-lasting changes in blood glucose and insulin levels [34]. Moreover, glucocorticoid signaling is important in fetal β-cell development. Increased glucocorticoids have shown to be associated with a decreased β-cell mass in rat pups [42]. Glucocorticoids stimulating PGC-1α and inhibiting Pdx-1 gene expression might lead to β-cells dysfunction [43]. In addition, glucocorticoids may influence obesity by blocking AMP-activated protein kinase and (in-)directly activating SREBP-1c gene expression. They may influence hypertension by imbalancing vasoactive factors [43].

### Adverse cardio-metabolic outcomes induced by maternal prenatal “stress”

Maternal stress that increases glucocorticoids is a known contributor for the development of obesity in the offspring [9, 18, 44]. Hence, glucocorticoid physiology has a potential impact on multiple targets of perinatal programming related to body composition and obesity risk [9].

Balasubramanian et al. revealed in an animal model study that prenatal maternal stress, that increases glucocorticoids, in combination with a high-fat diet increases the offspring’s risk for obesity in adulthood [44]. Moreover, prenatal stress (arising from socioeconomic or psychosocial factors that activate the HPA axis causing hypersecretion of cortisol) and a postnatal diet high in fat increases not only the offspring’s susceptibility to diet-induced obesity but also leads to secondary adverse metabolic consequences. Male and female offspring affected by hypersecretion of cortisol and a high-fat diet are similarly showing hyperleptinemia and hyperinsulinemia at weaning [45]. Moreover, several transgenic mouse models were developed to study the influence of increased maternal stress on adverse offspring health outcomes. The corticotropin-releasing factor receptor-2 deficient mouse is such a model of heightened stress sensitivity [46]. Chronic stress exposure (corticosterone levels) in these mice is linked to an increased intake of palatable foods. During exposure to chronic variable stress, transgenic mice consumed a greater proportion of their calories in form of a high-fat diet compared to non-stressed controls [47]. In transgenic mice selectively overexpressing 11β-HSD1 in adipose tissue, excess glucocorticoid exposure to the fetus produces visceral obesity. Moreover, these mice exhibited insulin resistance and diabetes [17].

In humans, maternal distress caused by flood exposure (self-reported psychological reaction to the flood) during an early phase of pregnancy was associated with a greater increase of the child’s BMI from age 2.5 to 4, as well as total adiposity at the age of 2.5. These results suggest that early gestation is a sensitive period for psychological maternal stress having a great impact on the development of childhood adiposity [48]. Furthermore, adolescents exposed to maternal psychosocial stress during intrauterine life consistently exhibit a higher BMI and body fat (%) with concomitant primary insulin resistance, and a lipid profile comparable to the metabolic syndrome [9]. Fetal exposure to glucocorticoids is able to program central adiposity in later life. Maternal corticotropin-releasing hormone levels found in the late second trimester of gestation, predicted child adiposity at 3 years of age. Thus, excess fetal exposure to glucocorticoids programs increased relative adiposity and central obesity in the offspring of prenatally stressed mothers [16].

Alterations in glucose-insulin metabolic function can also be induced by exposing the mother to excess glucocorticoid hormones. It is therefore possible that elevated circulating concentrations of stress hormones may elevate glucose levels in maternal and fetal circulation contributing to the development of insulin resistance [10].

Animal studies suggest that prenatal glucocorticoid exposure, either induced by hormonal treatment or by maternal stress during pregnancy, programs adverse metabolic alterations such as insulin resistance, hyperglycemia, and hyperinsulinemia [49, 50]. The aim of Lesage et al. (2004) was to investigate the consequences of prenatal stress (maternal glucocorticoids) on *in utero* growth restriction and a following increased risk to develop metabolic disorders [50]. Prenatal maternal stress reduced birth, adrenal and pancreas weight as well as plasma corticosterone and glucose levels in rat fetuses at term. In male rats 24 months of age, prenatal stress induced hyperglycemia and glucose intolerance. Adverse cardio-metabolic programming in the aged offspring could be linked to restricted fetal growth and elevated levels of circulating glucocorticoids [50].

Dancause et al. (2013) measured the effects of physiological prenatal stress caused by the Quebec Ice Storm in 1998 among a subsample of n = 32 exposed adolescents. Severity of stress was positively associated with insulin secretion, an early feature of insulin resistance suggesting that prenatal stress is an independent predictor of metabolic outcomes in adolescence [49]. Besides, physiological prenatal stress was associated with shorter length at birth and development of childhood adiposity which are predictors for an increased risk of cardio-metabolic diseases in adult life [49].

Furthermore, Igosheva et al. showed in an animal model study that stress exposure (a regimen of heat, light and restraint stress) early in development can have long-term effects on arterial blood pressure in adulthood, with potential sex differences [51]. Differences in cardiovascular function between prenatally stressed and control rats were especially evident when animals were challenged by acute restraint stress. In general, the detected effects were more obvious in female than in male animals [51]. In addition, van Dijk et al. revealed that prenatal maternal psychosocial stress predisposes for cardiovascular disease. Multiple psychosocial stressors during pregnancy were associated with higher systolic and diastolic blood pressure and mean arterial pressure in children (age 5–7) [19] and maternal depressive and anxiety symptoms were associated with lower offspring diastolic blood pressure and wider brachial artery diameter (age 10–12) [52].

## Discussion

In general, there are indications that maternal prenatal glucocorticoids play a key role in underlying mechanism in trans-generational programming of cardio-metabolic diseases. From a pathophysiological point of view, the maternal risk factor stress was clearly linked to adverse cardio-metabolic offspring outcomes such as obesity, insulin resistance, diabetes, metabolic syndrome, cardiovascular disease (hypertension), hyperglycemia, restricted fetal growth as well as reduced birth, adrenal, and pancreas weight (Fig 2).

The evaluation of the studies is affected by differences in the nature of the stress applied and its timing during pregnancy. Additionally, the effects of stress vary depending on the sex and age of the offspring [33].

In humans, prenatal maternal stress can be caused by exposure to both severe stressors and milder forms of psychosocial stress during pregnancy. Milder forms include stress experienced in daily life, but also pregnancy specific anxiety, e.g. the fear of giving birth to a handicapped child, or the mother suffering from prenatal depression [22].

Studies investigating the influence of maternal stress need to be carefully tested on potential confounding factors, which can cause the reported association to be misleading. Several maternal characteristics are considered to be confounders including age, educational level, ethnicity, tobacco and alcohol consumption [52].

Findings between studies can also vary considerably depending on the methodological approach used [11]. Some measures of maternal prenatal stress are based on mother’s self-reports, which might be biased by either over- or underreporting [20]. Maternal prenatal stress is converted into physiological signals that are subsequently transmitted to the fetus. By measuring clinical parameters, e.g. the mother’s heart rate or electrical conductance of the skin, it is possible to evaluate the maternal physiological arousal as a parameter of emotionality [20]. Descriptions of the children’s problems also often rely on a mother’s self-report, which makes it challenging to know whether the results simply show that anxious mothers characterize their children as being more difficult than non-anxious mothers do [20]. If human studies detect an association between physiological maternal prenatal stress and an adverse health outcome, the association may be mainly confounded by postnatal stress. Moreover, it can potentially be a result of shared genes or childrearing practices [20]. Since stress-related outcomes are likely dependent on a critical time-point during pregnancy, the timing of stress assessment is also an important factor in finding potential effects of prenatal stress on later disease development [28, 48]. Development of organs and tissues occurs over the course of gestation. Specific outcomes of stress-related perinatal programming are both dependent on the timing of stress exposure and the developmental stage of each individual organ system [34, 46]. Thus, vulnerable periods differ for the diverse outcomes because of different developmental stages of the various organs [22]. In that way, a mother’s stress experience during sensitive periods of cell and tissue growth may permanently alter the baby’s tissue structure and function, causing far-reaching effects which may persist throughout life [20].

Examples of extreme stressors are the exposure to (natural) disasters, e.g. earthquakes [22, 48]. These kind of disasters provide unique opportunities to investigate the effects of prenatal stress on offspring’s health outcomes independently from potential confounding factors. These disasters randomly affect women independent of their maternal and socioeconomic characteristics. Moreover, since the dates of such events are well-known, timing of stress exposure during pregnancy can be exactly determined [48].

Animal models are widely used to investigate the effect of maternal prenatal stress on a variety of different health outcomes in later life. A great advantage of laboratory animals is that stress responses can be reliably induced by a wide range of experimental methods. By exposing pregnant dams to stressful events (e.g. restraint) psychological stress is transferred to the fetus and thus produces effects on the offspring [20].

Even though the use of animal models provides benefits, substantial physiological differences between humans and animal models have to be taken into account [2, 33]. For example, the placental 11β-HSD2 expression declines towards term in mice, whereas it rises in humans. On this account the mouse might not be an ideal model of this biology [8]. Furthermore, the type of prenatal stress applied to animals is different to stressors affecting human pregnancy [20]. In animal studies, stressors are external events that can be controlled in terms of duration, frequency, and intensity [20, 45, 46], so that confounding factors can be successfully avoided [46]. Likewise, animal studies can be designed to make sure that the origin of stress-related effects is prenatal rather than postnatal, it is for example possible to cross-foster prenatally stressed pups to control dams after birth [33]. Compared to that, women who are psychologically stressed before pregnancy are more likely to be stressed postnatal too. Thus, it is difficult to distinguish social influences after birth from pregnancy effects that are transmitted biologically [20]. It also remains to be seen if an “optimal stress level” may beneficially affect offspring`s health [20].

It should also be mentioned that other uterine stress factors such as chronic stress, for example exposure to environmental contaminants, may or may not lead to an increased HPA axis, which can lead to cardiometabolic changes in the offspring [53].

Other systematic reviews and meta-analyzes support our findings. For instance, Lamichhane et al. (2019) [54] reported that there might be a direct association between psychological prenatal maternal stress and obesity in offspring. According to a meta-analysis by Burgueño et al. (2019) [55], prenatal maternal stress (increased release of glucocorticoids by activation of the HPA axis) was associated with increased BMI of their offspring. A meta-analysis by Lima et al. (2018) [56] found a significant association between antenatal stress exposure (adversely affected and hyperactivated HPA axis and sympathetic nervous system) and increased rates of low birth weight. Other reviews reported on the key role of glucocorticoids in fetal development and programming disease later in adult life [57, 58].

## Conclusions

This systematic scoping review provides an actual overview of (patho-) physiological mechanisms of the maternal risk factor stress on *in utero* programming and adverse health outcomes, and thus presents a basis for further research on specific mechanisms and individual outcomes. Although prenatal exposure to maternal stress clearly provoked adverse short- and long-term consequences in a broad range of animal and human studies, more research needs to be done to fully understand mechanisms by which maternal stress is leading to alterations in cardio-metabolic health outcomes.

## Supporting information

S1 Appendix PRISMA 2009 checklist. (DOC)

S2 Appendix Search strings. (DOCX)

S3 Appendix Overview of included studies. (DOCX)
