# Supplementary material for: Impact of maternal prenatal stress by glucocorticoids on metabolic and cardiovascular outcomes in their offspring: A systematic scoping review
Source: PLoS One. 2021 Jan 22;16(1):e0245386. doi: 10.1371/journal.pone.0245386 (PMC7822275; doi:10.1371/journal.pone.0245386)
Supplement: S2 Appendix — (DOCX) [file pone.0245386.s002.docx]

| ***MEDLINE (PubMed)*** | (((("Fetal Development"[Mesh]) OR (prenatal[Title/Abstract]) OR ("perinatal programming"[Title/Abstract] ) AND (((cortisol[MeSH Terms] OR (glucocorticoids[MeSH Terms]) OR (stress[Title/Abstract] ) AND (("Cardiovascular Diseases"[Mesh]) OR ("Metabolic Diseases"[Mesh]))) Filters: English, German, from 2000 – 2020 |
| --- | --- |
| ***EMBASE*** | (((("Fetal Development"/exp) OR (prenatal:ti,ab) OR ("perinatal programming”:ti,ab)) AND (((cortisol:ti,ab) OR (glucocorticoids/exp OR (stress/exp) AND (("Cardiovascular Disease"/exp OR ("Metabolic Disorder"/exp))) AND (([embase]/lim AND ([english]/lim OR [german]/lim) AND [2000-2020]/py)) |

Search strings.
