# Supplementary material for: Impact of maternal prenatal stress by glucocorticoids on metabolic and cardiovascular outcomes in their offspring: A systematic scoping review
Source: PLoS One. 2021 Jan 22;16(1):e0245386. doi: 10.1371/journal.pone.0245386 (PMC7822275; doi:10.1371/journal.pone.0245386)
Supplement: S3 Appendix — (DOCX) [file pone.0245386.s003.docx]

Overview of included studies.

| **Authors** | **Year** | **Topic** | **Human (H) or animal (A) study** | **Design** |
| --- | --- | --- | --- | --- |
| Rakers et al. | 2017 | Transfer of maternal psychosocial stress to the fetus | A/H | Review |
| Facchi et al. | 2020 | Perinatal programming of metabolic diseases: The role of glucocorticoids. | A/H | Review |
| Harris and Seckl | 2011 | Glucocorticoids, prenatal stress and the programming of disease | A/H | Review |
| Seckl and Holmes | 2007 | Mechanisms of Disease. Glucocorticoids, their placental metabolism and fetal 'programming' of adult pathophysiology | A/H | Review |
| Entringer et al. | 2012 | Fetal Programming of Body Composition, Obesity, and Metabolic Function. The Role of Intrauterine Stress and Stress Biology | A/H | Review |
| Monk et al. | 2012 | Linking prenatal maternal adversity to developmental outcomes in infants. The role of epigenetic pathways | A/H | Review |
| Painter et al. | 2012 | Long-term Effects of Prenatal Stress and Glucocorticoid Exposure | A/H | Review |
| Furukawa et al. | 2014 | A Comparison of the Histological Structure of the Placenta in Experimental Animals | A | Review |
| Zijlmans et al. | 2015 | Associations between maternal prenatal cortisol concentrations and child outcomes | A/H | Review |
| Seckl | 2004 | Prenatal glucocorticoids and long-term programming | A/H | Review |
| Reynolds | 2011 | Glucocorticoids and early life programming of cardiometabolic disease | A/H | Review |
| Seckl and Meaney | 2004 | Glucocorticoid Programming | A/H | Review |
| Glover et al. | 2010 | Prenatal stress and the programming of the HPA axis | A/H | Review |
| Drake et al. | 2007 | Mechanisms underlying the role of glucocorticoids in the early life programming of adult disease | A/H | Review |
| Cottrell | 2009 | Prenatal stress, glucocorticoids and the programming of adult disease | A/H | Review |
| Davis et al. | 2010 | The Timing of Prenatal Exposure to Maternal Cortisol and Psychosocial Stress Is Associated With Human Infant Cognitive Development | H | Cohort study |
| Ingstrup et al. | 2012 | Maternal Distress during Pregnancy and Offspring Childhood Overweight | H | Cohort study |
| Van Dijk et al. | 2012 | Prenatal Stress and Balance of the Child's Cardiac Autonomic Nervous System at Age 5-6 Years | H | Cohort study |
| Goedhart et al. | 2009 | Maternal cortisol and offspring birthweight | H | Cohort Study |
| Reynolds et al. | 2009 | Programming of Hypertension | H | Cohort Study |
| Dancause et al. | 2012 | Prenatal exposure to a natural disaster increases risk for obesity in 5½-year-old children | H | Cohort Study |
| Doyle et al. | 2000 | Antenatal corticosteroid therapy and blood pressure at 14 years of age in preterm children | H | Cohort Study |
| Dancause et al. | 2015 | Prenatal Stress due to a Natural Disaster Predicts Adiposity in Childhood | H | Cohort Study |
| Gillman et al. | 2006 | Maternal Corticotropin-Releasing Hormone Levels during Pregnancy and Offspring Adiposity | H | Cohort Study |
| Dancause et al. | 2013 | Prenatal stress due to a natural disaster predicts insulin secretion in adolescence | H | Cohort Study |
| Van Dijk et al. | 2012 | Association between prenatal psychosocial stress and blood pressure in the child at age 5-7 years | H | Cohort Study |
| Van Dijk et al. | 2014 | Association of maternal prenatal  psychosocial stress with vascular function in the child at age 10–11 years | H | Cohort Study |
| Entringer et al. | 2009 | Prenatal exposure to maternal psychosocial stress and HPA axis regulation in young adults | H | Non-randomized controlled trial |
| Entringer et al. | 2008 | Prenatal exposure to maternal psychosocial stress and HPA axis regulation in young adults | H | Non-randomized controlled trial |
| Dalziel et al. | 2005 | Cardiovascular risk factors after antenatal exposure to betamethasone | H | Randomized controlled trial |
| DiPietro | 2004 | Prenatal Maternal Stress in Child Development | H | Report |
| Kapoor et al. | 2006 | Fetal programming of hypothalamo-pituitary-adrenal function | H/A | Report |
| Masuzaki | 2001 | Transgenic Model of Visceral Obesity and the Metabolic Syndrome | A | Report |
| Lesage et al. | 2001 | Maternal Undernutrition during Late Gestation Induces Fetal Overexposure to Glucocorticoids and Intrauterine Growth Retardation, and Disturbs the Hypothalamo-Pituitary Adrenal Axis | A | Experimental study |
| Drake et al. | 2005 | Intergenerational consequences of fetal programming by in utero exposure to glucocorticoids in rats | A | Experimental study |
| Moritz et al. | 2011 | Prenatal glucocorticoid exposure in the sheep alters renal development in utero | A | Experimental study |
| Moritz et al. | 2009 | Haemodynamic characteristics of hypertension induced by prenatal cortisol exposure in sheep | A | Experimental study |
| Blondeau et al. | 2001 | Glucocorticoids impair fetal beta-cell development in rats | A | Experimental study |
| Balasubramanian et al. | 2015 | Differential effects of prenatal stress on metabolic programming in diet-induced obese and dietary-resistant rats | A | Experimental study |
| Tamashiro et al. | 2009 | Prenatal Stress or High-Fat Diet Increases Susceptibility to Diet-Induced Obesity in Rat Offspring | A | Experimental study |
| Mueller and Bale | 2006 | Impact of prenatal stress on long term body weight is dependent on timing and maternal sensitivity | A | Experimental study |
| Teegarden and Bale | 2008 | Effects of stress on dietary preference and intake are dependent on access and stress sensitivity | A | Experimental study |
| Lesage et al. | 2004 | Prenatal stress induces intrauterine growth restriction and programmes glucose intolerance and feeding behaviour disturbances in the aged rat | A | Experimental study |
| Igosheva et al. | 2004 | Prenatal stress alters cardiovascular responses in adult rats | A | Experimental study |
